# The trade-off between maximizing reconstruction and physiological interpretation of muscle synergies with autoencoders

**DOI:** 10.3389/fnhum.2025.1699799

**Published:** 2025-10-31

**Authors:** Cristina Brambilla, Nicol Moscatelli, Valentina Lanzani, Lorenzo Molinari Tosatti, Alessandro Brusaferri, Alessandro Scano

**Affiliations:** Institute of Intelligent Industrial Technologies and Systems for Advanced Manufacturing (STIIMA), Italian National Research Council (CNR), Milan, Italy

**Keywords:** muscle, synergies, autoencoder, non-negative, matrix, factorization, electromyography, accuracy

## Abstract

**Introduction:**

In neuroscience, the muscle synergy method is a widely known computational approach for studying motor control from electromyographic (EMG) recordings. Standard algorithms for synergy extraction rely on a linearity assumption for synergy combination. However, the interactions between muscle groups and movement dynamics often exhibit non-linear characteristics, suggesting the need for alternative approaches. In this context, autoencoders (AEs) have been proposed as promising tools. However, previous studies focused on the reconstruction accuracy optimization and not on the structure of the synergies, and the influence of AE design parameters has not been thoroughly investigated. This study aims to explore the impact of different activation functions on the effectiveness of AEs.

**Methods:**

To this end, we used a rich dataset of upper-limb EMG signals recorded from 16 muscles in 15 participants performing reaching movements toward 9 targets across 5 planes. We evaluated the effects of combining four activation functions in the encoder and decoder layers—linear, ReLU, sigmoid, and tanh—and compared to standard non-negative matrix factorization (NMF).

**Results:**

Our findings show that the extracted synergies are highly sensitive to the AE architecture. Notably, the configurations obtaining the best signal reconstruction do not correspond to the most physiologically meaningful synergies, which were instead achieved with the ReLU+tanh configuration.

**Discussion:**

This suggests that optimizing reconstruction accuracy may result in non-interpretable synergy structures. This research emphasizes the role of non-linear techniques in extracting muscle synergy from different datasets (e.g., lower limbs, full-body movements, patient populations) and identifies the optimal combination of transfer functions for the encoder and decoder layers.

## 1 Introduction

Muscle synergies are a computational framework used to explain how the central nervous system (CNS) simplifies motor control. Instead of activating each muscle independently, the CNS organizes movement through the coordinated activation of groups of muscles, or synergies (Bizzi et al., 2002). In this view, complex motor tasks are generated by combining a small set of synergies, each with a fixed pattern of muscle weights (spatial weights) modulated over time by activation coefficients (temporal components).

Traditionally, synergy analysis has been performed using linear factorization techniques of electromyographic (EMG) signals, such as non-negative matrix factorization (NMF) and principal component analysis (PCA), based on the assumption that muscle activations result from the linear combination of a limited number of synergies. Although this approach has proven to be effective in many contexts and derives from experiments on animals (Hart and Giszter, 2004; Kargo et al., 2010) and humans (Tresch et al., 2006), and the linearity assumption has been demonstrated to well reproduce experimental findings, non-linear approaches have not been rigorously tested with large and variable datasets. Given the inherently non-linear dynamics of the musculoskeletal system, including muscle activation-contraction coupling and joint biomechanics, it may be plausible that non-linear models might provide a more accurate and flexible description of motor control strategies (Cheung and Seki, 2021). In linear synergy models, the muscle activation matrix X is approximated as X ≈ WH, where W contains the synergy weights and H the activation coefficients. This formula assumes that the combination function is linear. However, non-linear models may even better capture the interactions between muscle groups and the dynamics of movement. For example, non-linear interactions may be especially relevant in clinical contexts, where abnormal coordination patterns emerge (Levin et al., 2000; Clark et al., 2010). Moreover, recent developments also aimed to connect muscle synergies to task space variables, such as kinematics or force outputs. Multi-domain relationships may contain some non-linearities. The mixed matrix factorization (MMF) algorithm, which linearly combines kinematic and muscular data into joint kinematic-muscular synergies (Scano et al., 2022), was introduced as a tool to link the synergistic domain with the task space. Other studies employed non-linear information-theoretic approaches to measure how much information about a task is encoded in each synergy (O’Reilly and Delis, 2024). These contributions underscore the relevance of non-linear models in advancing our understanding of sensorimotor integrations.

Recent research has begun to explore the use of non-linear machine learning methods—particularly artificial neural networks (ANNs)—in EMG signal processing. Among these, autoencoders (AEs) have emerged as a promising alternative for muscle synergy analysis. AEs consist of an encoder that compresses the input data into a low-dimensional representation, and a decoder that reconstructs the original input from this compressed form. Initial studies using AEs for muscle synergy analysis demonstrated that AEs could achieve a comparable reconstruction accuracy to standard linear methods such as NMF and PCA and capture the inhibition of agonist and antagonist muscles (Spüler et al., 2016). A convolutional AE was successfully applied in a study, demonstrating the effective reconstruction of EMG signals (Ding et al., 2021). In another investigation, Buongiorno et al. (2019) reported that AEs significantly outperformed NMF in terms of reconstruction accuracy during planar isometric force tasks. A comparative analysis of several factorization techniques showed that the AE outperformed factor analysis (FA) but not NMF (Zhao et al., 2022). In the context of clinical applications, Lee (2024) used an AE to extract muscle synergies from gait data, comparing healthy individuals and stroke patients. AEs have also been applied to extract kinematic synergies with a comparable performance to the PCA (De Feudis et al., 2021). A recent work conducted a systematic analysis on the use of AEs to extract muscle synergies (Giraud et al., 2025) and concluded that NMF and AE have similar performances; in specific setups (upper-limb workplace sectors), AE might perform slightly better.

The use of AEs in muscle synergy analysis remains limited, and existing studies can be improved in terms of clarification of the optimal setup for AEs. In general, the performance of the AE on synergy extraction has still been poorly investigated. First, no systematic investigation of the activation functions of the encoder and decoder layers has been conducted. Then, most studies focus on the quality of the reconstruction, but only a few directly assess synergy composition. Indeed, even when good reconstruction accuracy is achieved, synergies may not correctly reflect real neuromuscular modular organization, losing their physiological significance. Indeed, most of the previous studies did not compare in detail the synergy compositions between AE and NMF. The optimization of these parameters may be one of the fundamental steps for improving the performance and identifying the AE configuration that reconstructs the synergies underlying motor control.

Following these considerations, we performed a comprehensive evaluation of AEs for synergy extraction using a comprehensive dataset of multi-directional upper-limb reaching movements, combining four activation functions (linear, rectified linear unit—ReLU, sigmoid, and hyperbolic tangent). The dataset includes EMG signals from 16 muscles from 15 healthy subjects. Sixteen AE architectures were evaluated, defined by different combinations of encoder and decoder transfer functions. The AE performance was compared to the NMF using both reconstruction metrics (RMSE, R^2^) and synergy similarity metrics (cosine similarity for spatial weights and Pearson’s correlation for temporal activations). Our aim is to identify the most effective AE configuration for EMG decomposition and to explore the feasibility of using AEs as a flexible, non-linear alternative to linear synergy models.

The main contributions of our study are:

i)    the systematic comparison of autoencoder architectures based on all the possible combinations of 4 commonly employed transfer functions (16 total combinations).
ii)    the assessment of the performance of the AE, comparing reconstruction accuracy, synergy composition, and temporal components.
iii)    the use of a comprehensive upper limb dataset featuring movements in wide portions of the workspace.

## 2 Materials and methods

### 2.1 Participants

Fifteen healthy volunteers (42 ± 18 years old) participated in the study. Eligibility criteria required a full joint range of motion, absence of chronic or current musculoskeletal pain, no orthopedic impairments, and no history of musculoskeletal or neurological diseases. Individuals reporting acute injury, persistent pain, neurological symptoms, or current use of medication affecting neuromuscular function were excluded. The research was conducted at the Human Motion Analysis Laboratory, Consiglio Nazionale delle Ricerche (CNR—Lecco, Italy). Ethical approval was obtained from the CNR Ethical Committee (Rome, Italy). Before participation, all subjects provided written informed consent, and the study was conducted in accordance with the Declaration of Helsinki.

### 2.2 Experimental setup

The experimental setup was previously detailed in Scano et al. (2019). In summary, participants completed a point-to-point task, which involved reaching nine targets arranged on a board, starting from a reference position (R) located near the subject’s thigh in a comfortable position. Eight targets were positioned along a circumference of a 0.6 meter diameter circle at the cardinal and intercardinal directions [north (N), north-east (NE), east (E), south-east (SE), south (S), south-west (SW), west (W), north-west (NW)], while the 9th target (O) was placed at the center of the circle, following established protocols (d’Avella et al., 2006). Participants moved from R to each target and then returned to R. To comprehensively map the upper limb workspace, the target board was repositioned in five distinct locations relative to the participant: Frontal, Right, Left, upward (Up), and downward (Horizontal), ensuring a wide range of movement variability. For each orientation, ten trials of the reaching task were performed.

Kinematic data were collected using the Vicon motion capture system (Oxford, United Kingdom). Five reflective markers were placed on anatomical landmarks: the fifth dorsal vertebra (D5), seventh cervical vertebra (C7), acromion (representing the shoulder—S), right lateral elbow epicondyle (E), and ulnar styloid process (W). Participants held a 20-cm pointer equipped with two additional markers (EE1 and EE2). Movement onset and offset were determined using the velocity profile of the wrist marker. To emphasize phasic (dynamic) muscle activity in the EMG recordings, participants were instructed to perform the movements quickly. Surface EMG was recorded using 16 sEMG electrodes (Cometa, Milan, Italy), placed according to the SENIAM guidelines (Hermens et al., 2000), when available, on the following muscles: erector spinae (ES), teres major (TM), infraspinatus (IF), lower trapezius (LT), middle trapezius (MT), upper trapezius (UT), deltoid anterior (DA), deltoid middle (DM), deltoid posterior (DP), pectoralis (PT), triceps long head (TL), triceps lateral head (TLa), biceps long head (BL), biceps short head (BS), pronator teres (PR), and brachioradialis (BR).

### 2.3 EMG processing

Kinematic data were processed using the upper-limb model and a custom target model within the VICON Nexus System. Then, analysis of both kinematic and EMG data was performed in Matlab 2022 (Natick, USA), while muscle synergy extraction through AE was conducted in Python. EMG signals were filtered with a Butterworth 20–450 Hz bandpass filter, full-wave rectified, and filtered with a 6 Hz low-pass Butterworth filter. Movement phases were identified based on kinematic data as the movement from R to each target. To fully capture EMG activity, including electromechanical delay, a 200-ms interval was added before the movement onset and after the movement offset (Begovic et al., 2014). Only the phasic (motion-related) component of the EMG was analyzed. The tonic component was modeled as a linear ramp, interpolating between constant activation levels estimated from the average EMG activity 200 ms before movement onset and 200 ms after movement completion (d’Avella et al., 2006). The phasic EMG was obtained by subtracting this tonic component from the total EMG signal, with any resulting negative values set to zero. Each movement phase was temporally normalized to 100 equally spaced time points. All repetitions across all movement directions were concatenated into a single matrix. EMG channels were normalized to the maximum amplitude observed for each respective muscle across all trials. After preprocessing, the dataset was divided into training and testing subsets. The training set included data from nine out of ten trials for each point-to-point movement, while the remaining trial was allocated to the testing set. This data split follows standard practice in training autoencoders (Qian et al., 2020; Salehi et al., 2021).

### 2.4 Synergy extraction

An autoencoder is a type of neural network that implements two main architectures: an encoder that maps the input data *X* to a latent representation *h* (Equation 1), and a decoder that reconstructs the input into *X’* from this latent code (Equation 2).

These transformations are mathematically defined as follows:


(1) $$h=a\left(w_{1}\cdot X+b_{1}\right)$$

(2) $$X^{\prime }=w_{2}\cdot h+b_{2}$$

Equation 2 parallels the muscle synergy model, wherein the hidden representation *h* corresponds to the synergy activation coefficients, and the decoder weights *w*_2_ represent the muscle synergy vectors. The architecture of the AE followed the configuration proposed in previous studies (Buongiorno et al., 2020; Giraud et al., 2025), employing a single hidden layer with a number of neurons equal to the number of muscle synergies to be extracted. Four and six neurons were set for the hidden layer.

**FIGURE 1:**
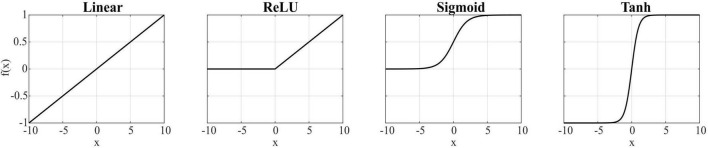
Activation functions used for the encoder and decoder layers.

The model was implemented in Python using the Keras library, based on the toolbox^1^ (Giraud et al., 2025) with the following hyperparameters: learning rate = 0.001; epochs number = 2,000; optimizer = *RMSprop*; loss function = mean squared error (MSE). These values were chosen to balance convergence quality and overfitting prevention. Additionally, the bias terms in both the encoder and decoder layers were removed to enhance performance, and the decoder weights were constrained to be positive (as muscle synergies are). Four different activation functions (linear, rectified linear unit (ReLU), sigmoid, and hyperbolic tangent) were evaluated and combined for the encoder and decoder layers, and the resulting solutions were compared. Chosen functions were linear, rectified linear unit (ReLU), sigmoid, and hyperbolic tangent (tanh), as shown in Figure 1. It should be noted that when using encoder activation functions that allow negative values and without positivity constraints, such as linear and tanh, negative temporal components may appear, which are physiologically unrealistic; however, we explored this scenario to assess the impact on the learned representations. The AE was trained using concatenated EMG data from all movement planes, learning a unified synergy representation across the full workspace. The AE was then tested for each plane, reproducing the multiple-plane architecture by Giraud et al. (2025).

For comparison, muscle synergies were also extracted using the NMF algorithm implemented in Python with the Coordinate Descent solver. The decomposition was iterated with randomly initialized matrices until the Frobenius norm between the original and the reconstructed matrices fell below 10^–4^, or a maximum of 500 iterations was reached. Four-synergy and six-synergy solutions were tested, and to mitigate the risk of local minima, 20 repetitions of the decompositions were performed on the test dataset. The solution with the lowest reconstruction error was selected for analysis.

### 2.5 Outcome measures and statistics

To evaluate the performance of each AE, the root mean square error (RMSE) and the reconstruction accuracy R^2^ were computed. The quality of reconstruction R^2^ of the original signal was defined as 1—SSE/SST, where SSE is the sum of the squared residuals, and SST is the sum of the squared differences with the mean EMG vector. Both RMSE and R^2^ are commonly used both in synergy analysis and in neural network training to quantify how well the input signal is reproduced, thus providing a direct assessment of the model performance. Additionally, the similarity between matched synergies extracted by each configuration and the one extracted by NMF was assessed using cosine similarity. Correspondence between the temporal activation coefficients of matched synergies was evaluated using Pearson’s correlation coefficient. These measures are crucial to assess whether the extracted synergies remain physiologically meaningful. Since NMF-derived synergies are considered as physiologically interpretable, comparing AE-based synergies against them allows to evaluate not only the reconstruction quality but also the preservation of physiologically meaningful structure in both spatial and temporal domains. Finally, the sparsity index of the spatial synergies was computed as the ratio between the number of zero-valued elements and the total number of elements in the synergy vector.

The statistical evaluation of AE’s performance was carried out through repeated measures ANOVA, with the AE configuration as within-subject factor. In case of significant main effect, pairwise post-hoc comparisons with Holm correction were performed to identify which AE configurations differed significantly.

## 3 Results

**FIGURE 2:**
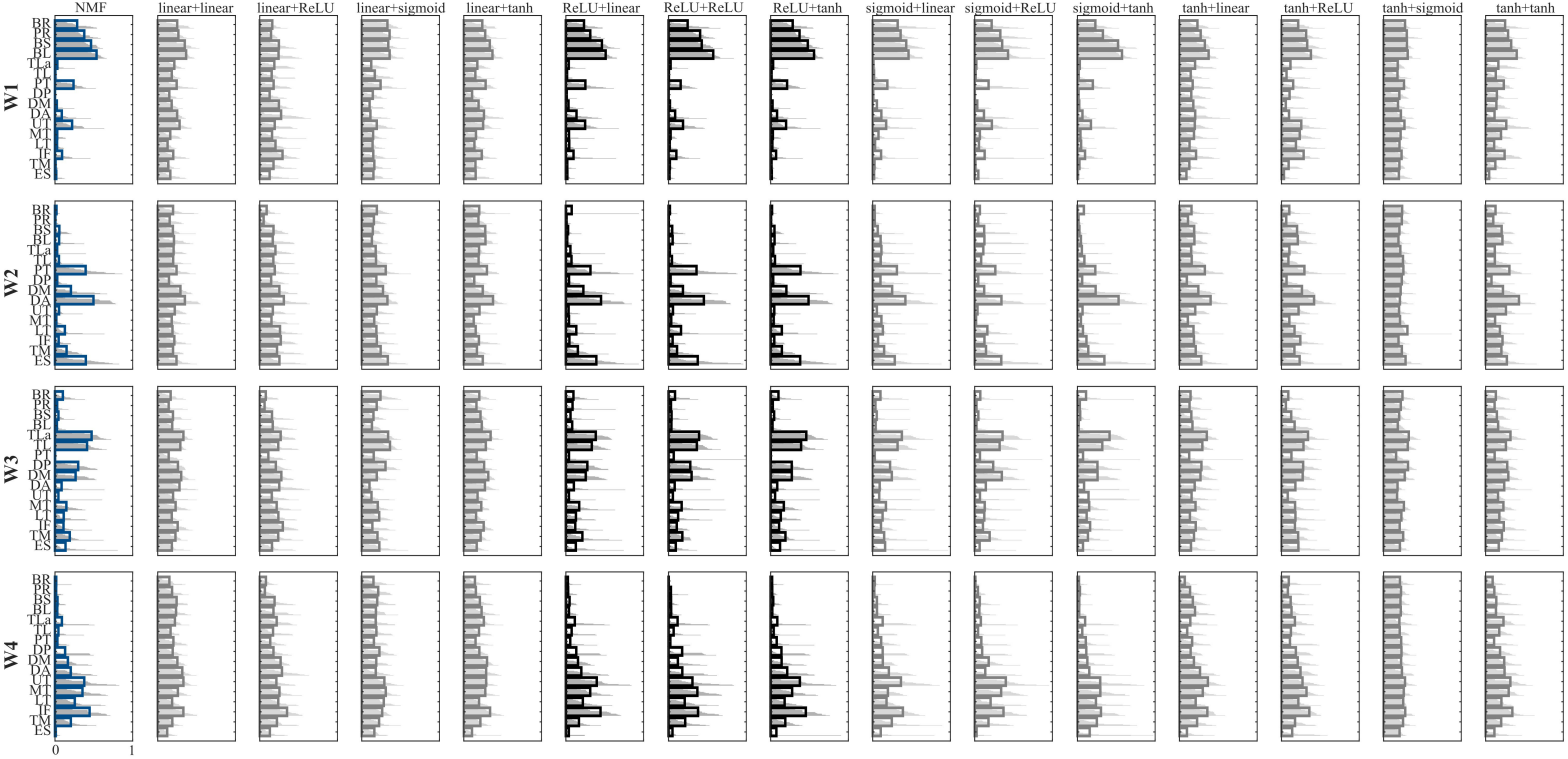
Comparison between the extracted synergies. Configurations are reported in the columns, while synergies are reported in the rows. Bold lines represent the mean value, while the light gray lines represent each participant. NMF synergies are reported in blue, while the configurations with the highest similarity to the NMF-based synergies are in black. In the label, the first term represents the encoder function, while the second term represents the decoder function.

**FIGURE 3:**
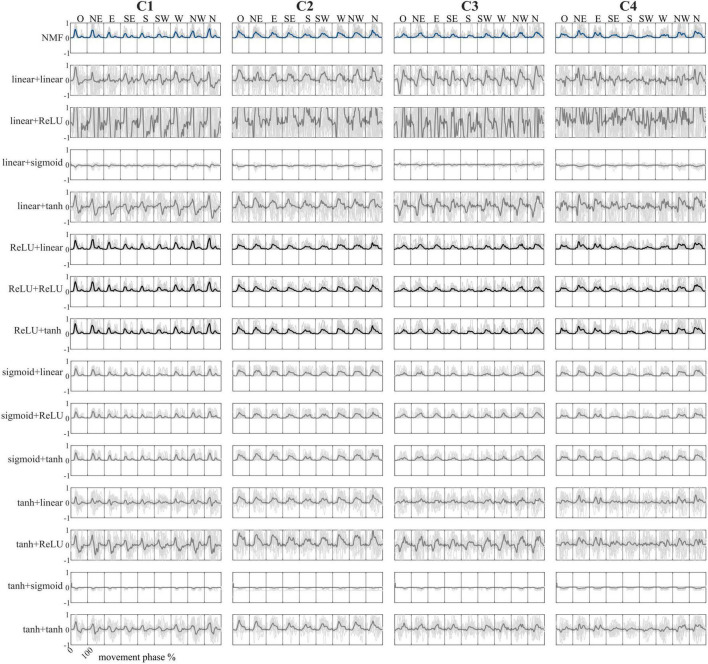
Comparison between the temporal coefficients in the frontal plane for all nine directions. Configurations are reported in the rows, while temporal coefficients are reported in the columns. Bold lines represent the mean value, while the light gray lines represent the average activation of each participant. NMF temporal coefficients are reported in blue, while the configurations with the highest correlations to the NMF-based temporal coefficients are in black. In the combination names, the first term represents the encoder function, while the second term represents the decoder function.

The mean spatial synergies (W) across participants extracted with NMF and all the AE configurations are shown in Figure 2, while the mean temporal coefficients reported for the frontal plane only are shown in Figure 3. Two combinations of AE (ReLU+sigmoid and sigmoid+sigmoid) are not analyzed because the temporal coefficients resulted in NaN. Further remedies for numerical instabilities, such as gradient clipping and initialization strategies, were adopted, but the reconstruction accuracy was negative, indicating that these combinations are not suitable for our analysis.

In the muscle synergies extracted using NMF, synergy W1 is primarily characterized by strong activation of the forearm muscles (BR and PR) alongside the biceps, with moderate contributions from pectoralis and UT. This synergy flexes the elbow at the beginning of the movement and controls the forearm at the end. Synergy W2 involves the activation of pectoralis, ES, DA, and DM supporting shoulder elevation during the reaching phase. Synergy W3 is dominated by the triceps and deltoids (mainly DP and DM), with contributions from the trapezii, reflecting a role in elbow extension. Synergy W4 mainly recruits the trapezii and back muscles, suggesting involvement in trunk stabilization and scapular control throughout the movement.

When comparing muscle synergies derived from AE to those from NMF, six AE configurations produced synergies closely resembling the NMF results. In contrast, the remaining AE combinations yielded synergies with widespread, non-specific activation across all muscles, reducing interpretability and functional relevance.

Regarding the NMF temporal coefficients, C1 is predominantly active at the beginning of the movement, reflecting initiation and forearm stabilization. C2 shows a peak midway through the movement, especially in upper directions, and is associated with shoulder flexion. C3 is activated toward the end of the motion, consistent with triceps involvement in elbow extension. Finally, C4 exhibits broad and sustained activation across all directions, indicating a role in continuous postural stabilization.

When comparing AE temporal coefficients to those from NMF, six AE configurations yielded comparable results. In contrast, combinations using a linear or hyperbolic tangent activation function in the encoder produced temporal coefficients with present negative values, which are incompatible with the non-negative structure of typical synergy models, and therefore may not be physiologically meaningful.

In Table 1, the results for all AE configurations are presented for the four-synergy solution. For NMF, RMSE was 0.073 (0.007) and R^2^ was 0.76 (0.03), with a sparsity index of 0.37 (0.04). The factor “AE configuration” was significant for all parameters (*p* < 0.001), indicating that some configurations performed significantly better or worse than others. The tanh+sigmoid configuration yielded the highest errors and lowest reconstruction (*p* < 0.03 compared to all other conditions). The best trconstruction accuracy was obtained with linear+linear, linear+tanh, tanh+linear, and tanh+tanh, although these differences were not significant relative to all the remaining configurations. In contrast, the highest similarity and correlation were observed for ReLU+tanh, ReLU+linear, and ReLU+tanh (*p* < 0.03 with respect to all the other configurations), which did not correspond to the most accurate reconstructions. The sparsity index was significantly higher in configurations using ReLU or sigmoid in the encoder (*p* < 0.001).

**TABLE 1:** Results for all AE configurations for the four-synergy solution.

Methods	RMSE	R^2^	Similarities	Correlations	Sparsity index
linear+linear	**0.072 (0.007)**	**0.76 (0.03)**	0.73 (0.05)	0.59 (0.12)	0.02 (0.02)
linear+ReLU	0.076 (0.011)	0.74 (0.06)	0.61 (0.05)	0.54 (0.10)	0.16 (0.06)
linear+sigmoid	0.080 (0.007)	0.71 (0.05)	0.75 (0.06)	−0.63 (0.09)	0.08 (0.03)
linear+tanh	**0.073 (0.007)**	**0.76 (0.04)**	0.72 (0.04)	0.56 (0.17)	0.02 (0.02)
ReLU+linear	0.073 (0.007)	0.75 (0.04)	**0.89 (0.11)**	**0.89 (0.11)**	**0.37 (0.04)**
ReLU+ReLU	0.084 (0.015)	0.68 (0.08)	**0.90 (0.10)**	**0.92 (0.09)**	**0.42 (0.07)**
ReLU+tanh	0.075 (0.007)	0.75 (0.04)	**0.96 (0.08)**	**0.96 (0.08)**	**0.35 (0.04)**
sigmoid+linear	0.079 (0.008)	0.72 (0.04)	0.86 (0.10)	0.83 (0.09)	**0.39 (0.04)**
sigmoid+ReLU	0.087 (0.010)	0.66 (0.08)	0.81 (0.12)	0.80 (0.11)	**0.44 (0.07)**
sigmoid+tanh	0.080 (0.009)	0.71 (0.04)	0.83 (0.10)	0.81 (0.08)	**0.39 (0.05)**
tanh+linear	**0.073 (0.007)**	**0.76 (0.03)**	0.79 (0.04)	0.67 (0.07)	0.13 (0.02)
tanh+ReLU	0.077 (0.009)	0.73 (0.06)	0.75 (0.06)	0.63 (0.07)	0.27 (0.05)
tanh+sigmoid	0.097 (0.009)	0.58 (0.08)	0.66 (0.03)	−0.39 (0.09)	0.02 (0.04)
tanh+tanh	**0.074 (0.007)**	**0.76 (0.03)**	0.78 (0.04)	0.64 (0.08)	0.12 (0.03)

RMSE, R^2^, sparsity index, similarities of synergies, and correlations of temporal coefficients of matched synergies between AE and NMF are reported. Standard deviations are reported in brackets. The highest values are reported in bold.

In Table 2, the results are shown for the six-synergy solution. For NMF, RMSE was 0.062 (0.007) and R^2^ was 0.83 (0.03), with a sparsity index of 0.51 (0.03). Again, the factor “AE configuration” was significant for each parameter (*p* < 0.001), with some configurations performing significantly better or worse. The tanh+sigmoid configuration resulted in the highest errors and lowest reconstruction accuracy (*p* < 0.002), while the best reconstruction accuracy was achieved with linear+ReLU, tanh+ReLU, and tanh+linear. Configurations including ReLU or sigmoid in the encoder showed the highest similarity and correlation (*p* < 0.01). The sparsity index was significantly higher in configurations using ReLU or sigmoid in the encoder (*p* < 0.001).

**TABLE 2:** Results for all AE configurations for the six-synergy solution.

Methods	RMSE	R^2^	Similarities	Correlations	Sparsity index
linear+linear	0.062 (0.006)	0.82 (0.04)	0.60 (0.04)	0.47 (0.10)	0.02 (0.01)
linear+ReLU	**0.058 (0.008)**	**0.85 (0.04)**	0.54 (0.04)	0.39 (0.22)	0.11 (0.03)
linear+sigmoid	0.071 (0.007)	0.77 (0.03)	0.67 (0.04)	−0.58 (0.08)	0.10 (0.01)
linear+tanh	0.062 (0.007)	0.82 (0.03)	0.60 (0.04)	0.38 (0.10)	0.02 (0.01)
ReLU+linear	0.065 (0.007)	0.81 (0.03)	**0.88 (0.09)**	**0.89 (0.09)**	**0.46 (0.05)**
ReLU+ReLU	0.065 (0.009)	0.81 (0.03)	0.82 (0.08)	0.83 (0.09)	**0.47 (0.04)**
ReLU+tanh	0.065 (0.008)	0.81 (0.04)	**0.86 (0.10)**	**0.87 (0.09)**	**0.47 (0.04)**
sigmoid+linear	0.072 (0.007)	0.77 (0.04)	0.82 (0.06)	0.81 (0.05)	**0.52 (0.04)**
sigmoid+ReLU	0.075 (0.010)	0.74 (0.07)	0.80 (0.07)	0.78 (0.09)	**0.54 (0.04)**
sigmoid+tanh	0.072 (0.007)	0.77 (0.03)	0.83 (0.09)	0.81 (0.09)	**0.52 (0.04)**
tanh+linear	**0.062 (0.007)**	**0.83 (0.03)**	0.70 (0.03)	0.57 (0.04)	0.14 (0.02)
tanh+ReLU	**0.060 (0.008)**	**0.84 (0.03)**	0.68 (0.05)	0.55 (0.06)	0.24 (0.04)
tanh+sigmoid	0.086 (0.008)	0.67 (0.04)	0.62 (0.03)	−0.46 (0.06)	0.05 (0.03)
tanh+tanh	0.064 (0.007)	0.82 (0.03)	0.70 (0.03)	0.55 (0.06)	0.14 (0.02)

RMSE, R^2^, sparsity index, similarities of synergies, and correlations of temporal coefficients of matched synergies between AE and NMF are reported. Standard deviations are reported in brackets. The highest values are reported in bold.

## 4 Discussion

In this study, multiple AE architectures for muscle synergy extraction have been investigated, focusing on the influence of encoder and decoder activation functions. Our results show that specific configurations can effectively approximate the performance of linear NMF, both in terms of EMG reconstruction accuracy and synergy interpretability. Notably, six AE configurations demonstrated synergy patterns and temporal activations closely resembling those obtained from NMF, highlighting the potential of AEs as feasible non-linear alternatives for muscle synergy extraction.

### 4.1 AE configurations and physiological interpretability

The most used activation functions were systematically tested in all possible encoder-decoder combinations. This choice was motivated by the fact that activation functions influence the resulting synergies, although their impact has not been systematically investigated in previous studies. Moreover, earlier work primarily emphasized reconstruction accuracy, often overlooking whether the extracted synergies were physiologically meaningful. Indeed, a high reconstruction R^2^ does not necessarily guarantee physiologically valid synergies. The choice of activation functions in both the encoder and decoder influences the quality and interpretability of the extracted synergies. Configurations employing ReLU or sigmoid for the encoder layer generally produced physiologically meaningful synergies, preserving spatial specificity and muscle grouping consistent with NMF.

Conversely, configurations with linear or hyperbolic tangent functions in the encoder often resulted in widespread, non-selective muscle activations associated with negative temporal coefficients, which are inconsistent with the synergy model and compromise physiological validity, as negative coefficients have no physiological interpretation in standard synergy models. When using linear or tanh, the temporal coefficients include negative values, as expected, because these functions allow both positive and negative outputs. The specificity of the extracted synergies is reduced, as negative coefficients enable the model to reconstruct EMG activity by both subtracting and adding synergies, effectively increasing the flexibility of the decomposition. While this may improve the reconstruction accuracy, it leads to spatial synergies that are less selective, often involving widespread co-activation of many muscles. Consequently, the resulting synergies lack the spatial specificity and interpretability typically expected in standard synergy models.

Using ReLU in the encoder combined with a sigmoid decoder, or sigmoid functions in both encoder and decoder, led to NaN values in the temporal coefficients. This behavior can be attributed to numerical instabilities. In the first case (ReLU+sigmoid), the unbonded positive outputs of the ReLU can drive the sigmoid activation in the decoder into saturation, resulting in extreme gradients or numerical overflow. In the second case (sigmoid+sigmoid), stacking sigmoids in both the encoder and decoder increases the risk of saturation at either end of the activation range, which leads to vanishing gradients and unstable weight updates. Both scenarios disrupt the training dynamics and ultimately cause the coefficients to diverge to NaN. Additional remedies for improving their performance were employed, such as gradient clipping and initialization strategies, but the R^2^ became negative, indicating that the reconstruction error was larger than the signal variance. These combinations are not suitable for the present architecture, and an exhaustive hyperparameter tuning or architecture redesign for these two combinations is outside the scope of the present study.

In line with previous works (Buongiorno et al., 2019; Giraud et al., 2025), our results confirm that AEs can match or slightly outperform NMF in terms of EMG reconstruction metrics. However, similar performance in reconstruction does not guarantee equivalent synergy structure, a distinction often overlooked, or at least not clarified, in prior studies. Our analysis directly addressed this by evaluating spatial (cosine similarity) and temporal (Pearson’s correlation) similarity between AE- and NMF-derived synergies, revealing that only a subset of AE configurations maintains the physiological coherence of muscle co-activations, even when reconstruction metrics are solid. Our conclusions remained consistent at both 4-synergy and 6-synergy solutions. Indeed, our findings highlighted an important distinction between reconstruction accuracy and physiological interpretability. Linear autoencoders, in particular, often reach high R^2^ values, reflecting strong linear contributions to the reconstruction of EMG signals. However, such high reconstruction performance does not necessarily guarantee that the extracted components correspond to physiologically meaningful synergies, such as in the case of a linear activation function in the encoder layer. Rather, it may simply indicate that the model is efficiently capturing the variance in the data through linear combinations. Therefore, while linear AEs may serve as powerful tools for data compression and reconstruction, their outputs should be interpreted with caution in physiological contexts.

### 4.2 Advantages and challenges of AE-based synergy extraction

AEs offer several advantages over linear methods. Their flexibility allows modeling complex, non-linear dependencies in muscle activations, which may even better reflect the underlying biomechanics and neurophysiology of the movement with respect to linear models (which are already deemed as effective to model standard combinations of synergies). Moreover, it could be interesting to explore the application of AE-based methods in other domains that may exhibit non-linear characteristics, such as task space (Scano et al., 2022) or torque space (Russo et al., 2014), in which the contributions of non-linear models might be crucial. This is particularly relevant in clinical settings, where a more accurate representation of the relationship between muscle activity and task execution can enhance diagnostic and therapeutic approaches. For instance, the MMF algorithm, which extracts kinematic-muscular synergies, assumes a linear mapping between kinematic and muscular spaces; however, this simplification may not fully capture the underlying dynamics (Scano et al., 2022). Additionally, research using non-linear models to assess the informal content of synergies in task performance has shown that these models can detect motor impairments that standard NMF-based methods do not capture (O’Reilly and Delis, 2024). Future work will focus on applying AEs to extract synergies across multiple domains characterized by non-linear interactions, to evaluate whether this approach surpasses traditional linear techniques. Establishing robust links between muscle activity and task space can support the development of assistive rehabilitation technologies, such as exoskeletons and end-effector robots, by leveraging kinematic-muscular synergies that integrate both motor command and movement output.

### 4.3 Clinical and research applications

Given their ability to model non-linear relationships, AEs show strong potential for applications in rehabilitation, particularly for patient populations with atypical coordination patterns (e.g., stroke). The adaptability of AE architectures may allow detection of subtle deviations in synergy structure that linear models might miss, potentially offering biomarkers for diagnosis, therapy planning, or motor recovery assessment. Moreover, the success of AEs in healthy participants supports their use as a tool for understanding normative motor control strategies. With proper tuning, AEs could also be instrumental in tasks involving robotic assistance, prosthesis control, or biofeedback training. Non-linear synergy models may reveal motor control deficits that may not be detected when using linear approaches. In fact, motor impairments might induce non-linearities in the neuromuscular system that are absent in healthy individuals. Moreover, establishing a direct link between muscle activation and task space is particularly valuable in clinical contexts. Understanding how neural drive translates to motor outcomes can help identify intact synergies that are deployed in biomechanically suboptimal ways. This distinction could guide rehabilitation strategies that not only aim to restore normal synergy patterns but also enhance their functional implementation during task performance. While non-linear models hold considerable clinical promise, they typically require large datasets with many movement repetitions to ensure reliable training and generalization. This practical consideration should be taken into account when designing future clinical studies or therapeutic protocols based on these methods.

### 4.4 Limitations and future directions

Our findings also expose limitations. The AE’s performance is sensitive to architectural choices such as transfer functions, and our conclusions are specific to the dataset used. To assess the generalizability of AE-based synergy extraction, future studies should involve different datasets, such as those involving lower limb movements or hand grasps. This will help determine whether AEs can provide more robust or complementary insights compared to NMF, and whether the assumption of linearity holds across different muscle groups. Previous research has shown that both the selected task and muscle set can significantly influence the resulting synergies, revealing novel intermuscular relationships (Steele et al., 2013; Brambilla and Scano, 2022). Therefore, validating AE performance across multiple datasets is a crucial next step. Additionally, this study employed a relatively simple AE architecture, with a single hidden layer and the number of synergies fixed at four. Future work should explore more diverse architectures, including deeper networks and varying numbers of synergies, to better understand how these factors influence performance. Extending the analysis to pathological populations could also offer important insights into the clinical applicability of AE-based models.

Interestingly, while the extracted synergies were similar to the NMF in some combinations, variations depending on AE settings suggest that one approach may outperform the others. However, due to the presence of noise in EMG signals, it is currently not possible to definitively establish which method produces superior results. One promising strategy for benchmarking performance would be to use simulated ground truth synergies and temporal coefficients defined *a priori* by the experimenter. By multiplying these to generate synthetic EMG envelopes, one could then apply NMF and the best combination of AE to extract synergies and quantitatively compare their accuracy. The success of this approach depends on designing physiologically plausible ground truth datasets, a challenge that future work will address, following methodologies similar to those proposed by Scano et al. (2022).

## 5 Conclusion

This study systematically evaluated AE architectures for muscle synergy extraction, focusing on activation function combinations. We found that while some configurations offered high reconstruction accuracy, they did not always produce physiologically meaningful synergies. The ReLU+tanh setup provided the most similar synergies to the NMF. These findings highlight the importance of selecting appropriate activation functions when using AEs for EMG analysis and suggest that non-linear methods can capture motor control features missed by linear models. Future research should explore AE applications in more complex or clinical datasets.

## Data Availability

The raw data supporting the conclusions of this article will be made available by the authors, on reasonable request and with ethical authorization.

## Footnotes

1
https://github.com/cbrambilla/musclesynergyextractionbench-main
